# Elevated expression of TLR2 and its correlation with disease activity and clinical manifestations in adult-onset Still’s disease

**DOI:** 10.1038/s41598-022-14004-4

**Published:** 2022-06-17

**Authors:** Jae Ho Han, Mi-Hyun Ahn, Ju-Yang Jung, Ji-Won Kim, Chang-Hee Suh, Ji Eun Kwon, Hyunee Yim, Hyoun-Ah Kim

**Affiliations:** 1 Department of Pathology, Ajou University School of Medicine, Suwon, Korea; 2 Department of Rheumatology, Ajou University School of Medicine, 164 Worldcup-ro, Yeongtong-gu, Suwon, 16499 Korea

**Keywords:** Biomarkers, Rheumatology

## Abstract

This study investigated the role of Toll-like receptor 1 (TLR1), TLR2, TLR4, TLR7, and TLR9 in patients with adult-onset Still’s disease (AOSD). This study included 20 patients with AOSD and 15 healthy controls (HCs). TLR expression in the peripheral blood was quantified using flow cytometry; TLR expression pattern, in the skin lesions and lymph nodes (LNs) of patients with AOSD, was evaluated immunohistochemically. Significantly higher mean intensities of cells presenting TLR2 and TLR7 from whole blood were observed in patients with AOSD than in HCs. TLR2 expression in whole cells correlated with systemic scores, levels of lactate dehydrogenase and ferritin and serum levels of interleukin-1β (IL-1β), IL-6, and IL-18. The percentage of TLR2-positive inflammatory cells was higher in skin biopsy samples from patients with AOSD than those in HCs. TLR9-expressing positive inflammatory cell counts were higher in skin lesions from patients with AOSD than those in the HC, eczema, and psoriasis groups. The expression levels of TLR1, TLR4, TLR7, and TLR9 were higher in LNs of patients with AOSD than in those with T cell lymphoma and reactive lymphadenopathy. Circulating TLR2- and TLR7-positive cells may contribute to the pathogenesis of AOSD. Furthermore, immunohistochemical staining for TLRs in skin lesions and LNs may aid in differentiating AOSD from similar conditions.

## Introduction

Adult-onset Still’s disease (AOSD) is a systemic autoinflammatory disorder characterized by spiking fever, evanescent skin rashes during fever, multiple arthralgia, organomegaly, and serositis^1^. Although the aetiology of AOSD is still inconclusive, several factors, such as genetic background, viral or bacterial infections, and aberrant immune response are involved. AOSD inflammation is triggered by the activation of the innate immune system along with aberrant production of proinflammatory cytokines, such as interleukin (IL)-1β, tumour necrosis factor-α (TNF-α), and IL-18^2, 3^.

Some clinical manifestations of AOSD, such as spiking fever, lymphadenitis, and liver enzyme elevation, are similar to those of viral or bacterial infections, suggesting that infection may trigger the initial inflammatory response in AOSD. Many viruses, including adenovirus, coxsackievirus, Epstein-Barr virus, echovirus 7, severe acute respiratory syndrome coronavirus 2, and certain bacteria are associated with the disease^4–13^. Environmental factors activate the innate immune system; subsequently, the pattern-recognition receptors (PRRs) on immune cells interact with pathogen-associated molecular patterns (PAMPs). PAMPs include viral single-stranded DNA or RNA or bacterial components such as lipopolysaccharides (LPS) in gram-negative bacteria. The association of damage-associated molecular patterns (DAMPs), that occur after various inflammations, with the pathogenesis of AOSD has also been reported^14^. Elevated levels of high mobility group box 1 (HMGB1), S100 protein, and histones have been reported in the sera of patients with acute AOSD^2, 15–17^.

Several PRR families, including C-type lectin receptors, Toll-like receptors (TLRs), and nucleotide-binding oligomerization domain receptors (NOD-like receptors, NLRs), recognize PAMPs and DAMPs in several infectious and inflammatory diseases^18^. TLRs are type I transmembrane proteins located in intracellular endosomes, plasma membranes, or both^19^. TLRs interact and respond to danger signals through intracellular signalling pathways and sense PAMPs and DAMPs in non-immune and immune cells^14^. TLRs transmit signals through specific sets of adaptors and transcription factors and trigger various cellular responses, resulting in the increased expression of inflammatory factors, such as type I interferon (IFN), chemokines, IL-1, and IL-6^18, 20^. TLR-mediated inflammation is implicated in the pathogenesis of sterile inflammations, such as wound healing, autoimmune or autoinflammatory diseases, and malignancies^20, 21^. Especially, TLR1, TLR2, TLR4, TLR7 and TLR9 have been evaluated in several rheumatic diseases and their putative pathological roles were shown^20^. Altered TLR ligands have also been implicated in the pathogenesis of AOSD, especially TLR4 and its ligands, such as S100 proteins and HMGB1^15, 16, 22^. The expression of TLR7 on circulating precursors of myeloid dendritic cells (pre-mDCs) and mDCs considerably increased in patients with AOSD compared to that of healthy controls (HCs); TLR7 transcription levels correlated with the serum IL-1 and IFN-α levels in AOSD^23^. However, there are limited data regarding the association of TLRs with the manifestations of AOSD in patients; the available data focus on TLR4 and TLR7^22, 23^. To the best of our knowledge, the expression of TLR1, TLR2, and TLR9 in patients with AOSD has not yet been evaluated.

Therefore, we investigated the potential role of TLR1, TLR2, TLR4, TLR7, and TLR9 in the pathogenesis of AOSD, and their levels were quantified in the peripheral blood of patients with AOSD and HCs using flow cytometry. Furthermore, we used immunohistochemistry to detect the expression of these markers and evaluated their levels in the skin and lymph nodes (LN) of patients with AOSD compared with those of other skin diseases.

## Results

### Clinical characteristics of the patients

Table 1 shows the clinical characteristics of the 20 patients with AOSD and 15 HCs. There was no significant difference in the age or sex of patients with AOSD and HCs. Nine were in the initial stages before treatment, and the disease duration of the remaining 11 patients was 84.3 (29.8) months. Among the 11 patients, four discontinued their medications before the flare. Three patients were treated with methotrexate and intravenous tocilizumab, three patients were treated with methotrexate and leflunomide, and one patient was treated with azathioprine, at the time of sampling. There were no patients diagnosed with macrophage activation syndrome or fulminant hepatitis. The daily glucocorticosteroid dose of the seven patients with AOSD was 3.2 (1.2) mg prednisolone equivalent.

**Table 1:** Clinical characteristics of patients.

	AOSD (n = 20)	HC (n = 15)	p-value
Age (years)	50.1 ± 13.6	43.2 ± 7.8	0.069
Gender (F/M)	13/7	7/8	0.321
Systemic manifestation/chronic articular manifestation	15/5		
Fever	10 (50)		
Sore throat	4 (20)		
Skin rash	12 (60)		
Lymphadenopathy	3 (15)		
Splenomegaly	2 (10)		
Hepatomegaly	2 (10)		
Pericarditis	0 (0)		
Arthritis	6 (30)		
Hemoglobin, g/dL	12.7 ± 1.7		
Leukocytes, /μL	9,515 ± 4,570		
Neutrophils, /μL	7,306 ± 4,447		
Platelets, × 10^3^/μL	287.7 ± 137.0		
Ferritin, ng/mL	2,137.6 ± 4,512.0		
ESR, mm/h	37.1 ± 31.8		
CRP, mg/dL	5.63 ± 9.06		
AST/ALT, mg/dL	39.7 ± 39.1/34.8 ± 27.4		
Bilirubin, mg/dL	0.55 ± 0.31		
Albumin, g/dL	4.2 ± 0.6		
Systemic score	2.5 ± 1.9		
IL-1β, pg/mL	1.86 ± 5.99	0.00 ± 0.00	0.214
IL-6, pg/mL	9.56 ± 15.1	0.00 ± 0.00	0.012
IL-18, ng/mL	7.47 ± 6.44	0.44 ± 0.34	< 0.001
TNF-α, pg/mL	4.54 ± 7.65	0.42 ± 1.25	0.086

AOSD, adult-onset Still’s disease; HC, healthy control; ESR, erythrocyte sedimentation rate; CRP, C-reactive protein; AST, aspartate transaminase; ALT, alanine transaminase; IL, interleukin; TNF, tumor necrosis factor. All values are presented as numbers (with percentages) or means ± standard deviations. The systemic scoring system of Pouchot et al.^40^ assigns a score from 0 to 12 with 1 point for each of the following manifestations: fever, typical rash, pleuritis, pneumonia, pericarditis, hepatomegaly or abnormal liver function test data, splenomegaly, lymphadenopathy, leukocytosis ≥ 15,000/mm^2^, sore throat, myalgia, and abdominal pain. These data were assessed using a Mann–Whitney U test or Fisher’s Exact test.

The IL-6 levels in AOSD patients [9.56 (15.1) pg/mL] were higher than those in HC [0.0 (0.0) pg/mL, p = 0.012]. Furthermore, the IL-18 levels in AOSD patients [7.47 (6.44) ng/mL] were higher than those in HC [0.44  (0.34) ng/mL, p < 0.001]. However, there was no statistically significant difference in the levels of IL-1β and TNF-α between AOSD patient and HCs, although those levels in AOSD were higher than those in HCs.

### Microarray data in patients with AOSD and HCs

**Figure 1:**
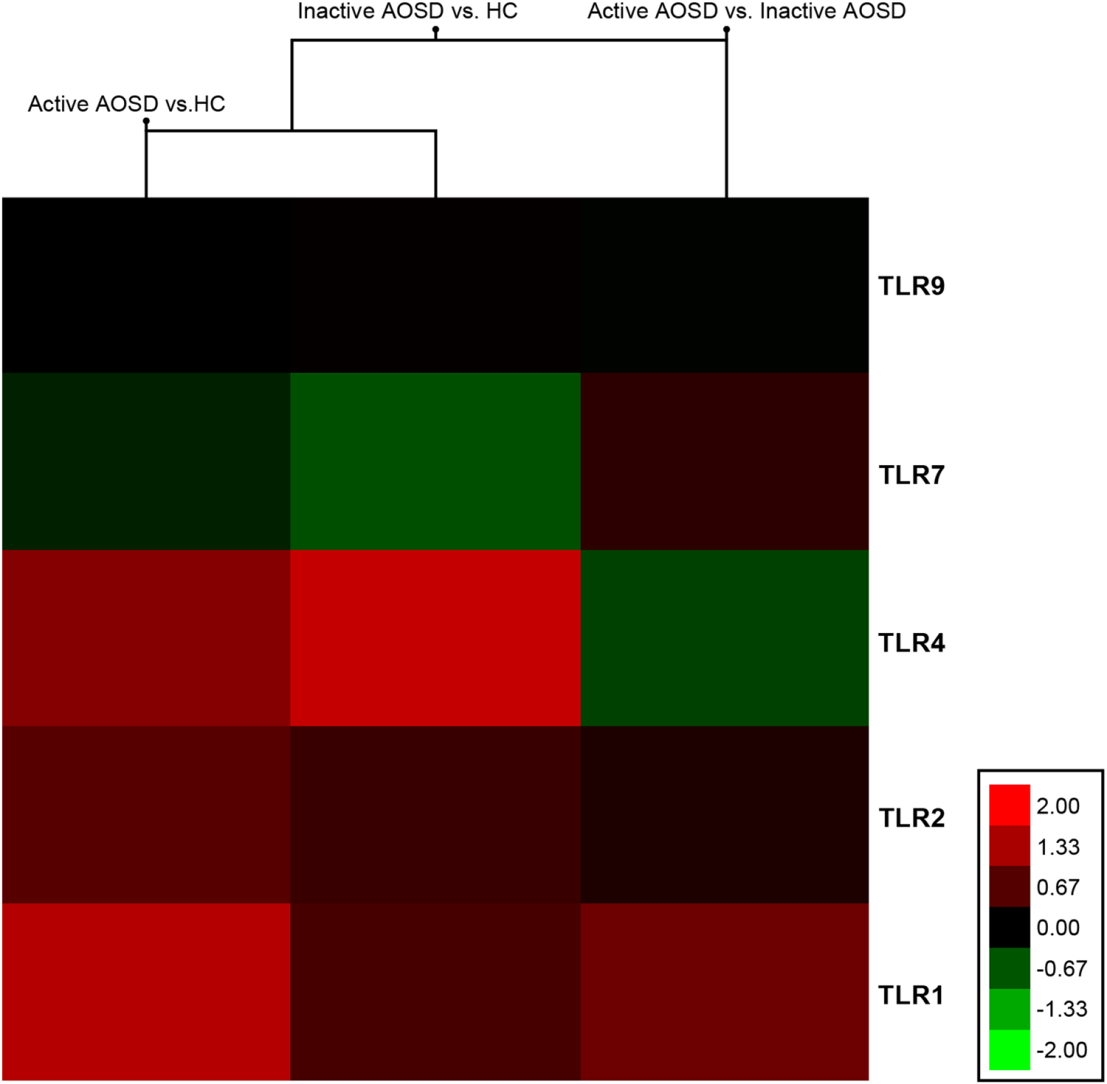
A heatmap of the toll-like receptor (TLR)1, TLR2, TLR4, TLR7, and TLR9 genes in peripheral blood mononuclear cells from active adult-onset Still’s disease (AOSD) relative to the healthy controls (HCs) or the inactive AOSD. The expression levels of TLR1, TLR2, and TLR4 in active AOSD or inactive AOSD were increased in comparison to those of the HC. However, the expression levels of TLR7 and TLR9 in active AOSD or inactive AOSD were not different to those of the HC. The expression levels of TLR1 and TLR7 in active AOSD were increased than those of inactive AOSD.

Microarray data analysis revealed significant changes in the expression of genes related to TLR signalling between the active and inactive AOSD groups, between active AOSD and HC, or between inactive AOSD and HC (adjusted p-value from LIMMA test < 0.05). Among the DE genes, 60 were upregulated, and 34 were downregulated. Ten genes were also found to be differentially expressed (adjusted p-values < 0.05) in active AOSD compared to HC. The lists of significant DE genes (in active AOSD vs. HC, inactive AOSD vs. HC, and active vs. inactive AOSD) were compared using Venn diagrams (Table S1). In particular, the expression levels of TLR1, TLR2, TLR4, TLR7, and TLR9 genes in active AOSD relative to the HC or inactive AOSD are shown as a heatmap (Fig. 1). The expression levels of TLR1, TLR2, and TLR4 in active AOSD or inactive AOSD were increased in comparison to those of the HC. However, the expression levels of TLR7 and TLR9 in active AOSD or inactive AOSD were not different to those of the HC. The expression levels of TLR1 and TLR7 in active AOSD were increased than those of inactive AOSD.

### The intensity of cells presenting TLR1, TLR2, TLR4, TLR7, and TLR9 in patients with AOSD and HCs

**Figure 2:**
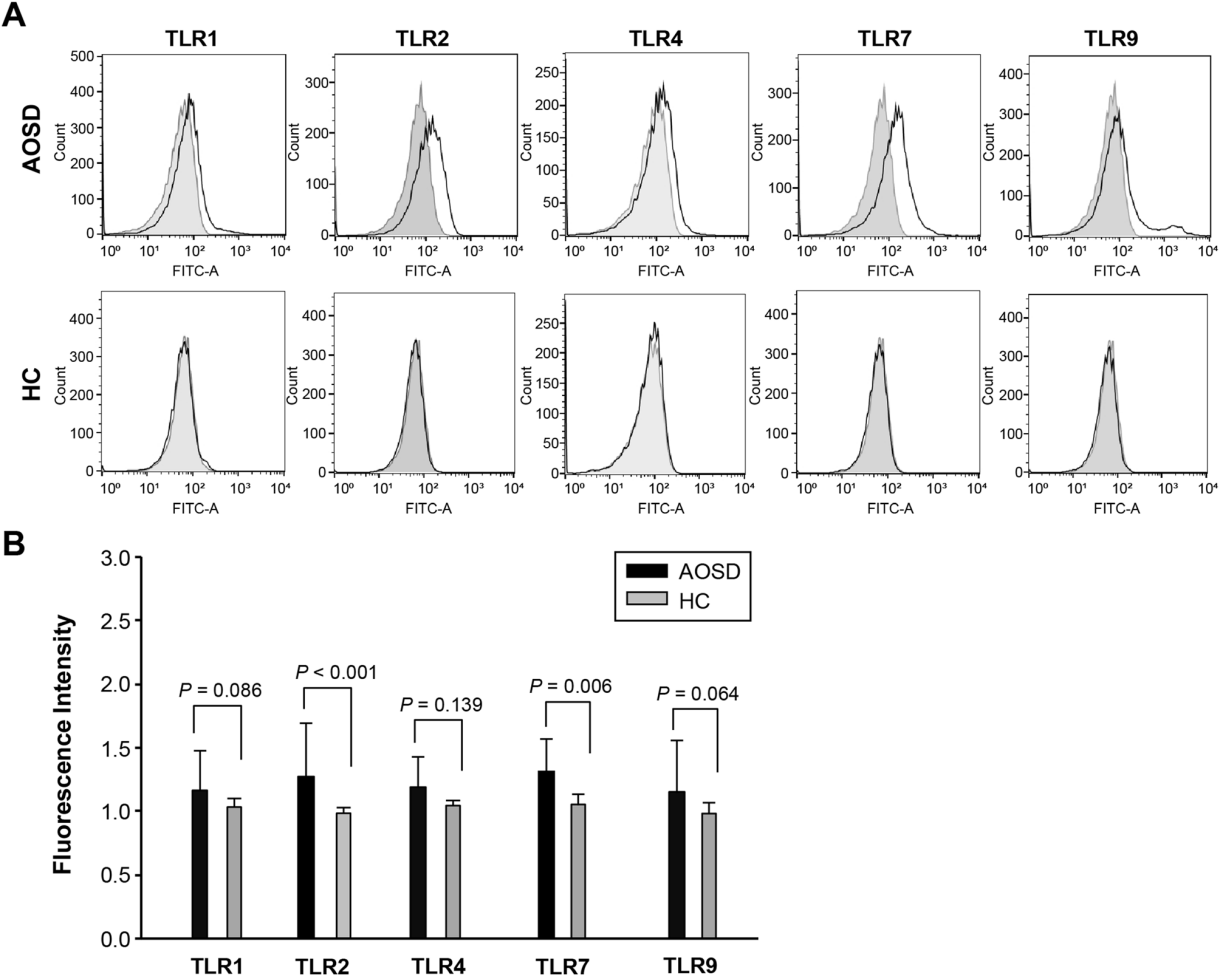
(**A**) Representative flow cytometric histograms of stained cells presenting toll-like receptor (TLR) from peripheral blood of one patient with adult-onset Still’s disease (AOSD) and a healthy control (HC) are shown. (**B**) Flow cytometric results of the percentage of surface-stained cells presenting TLR1, TLR2, TLR4, TLR7, and TLR9 in patients with AOSD and HC. Results were obtained from 20 patients with AOSD and 15 HCs. The bar with horizontal line indicates the mean value with standard deviation for each group. The p-value was determined using the Mann–Whitney *U*-test. Significantly higher mean intensity of stained cells presenting TLR2 was observed in patients with AOSD than in HC (p < 0.001). A significantly higher intensity of cells presenting TLR7 from whole blood was seen in patients with AOSD than in HC (p = 0.006). However, there was no significant difference in the intensity of cells presenting TLR1, TLR4, or TLR9 between patients with AOSD and HCs.

Representative examples of flow cytometric histograms of cells presenting several TLR markers from the peripheral blood of one patient with AOSD and an HC are shown in Fig. 2. Significantly higher mean intensity of cells presenting TLR2 from whole blood was observed in patients with AOSD [1.27 (0.43)] than in HC [0.98 (0.04), p < 0.001]. A significantly higher intensity of cells presenting TLR7 from whole blood was seen in patients with AOSD [1.28 (0.29)] than in HC [1.06 (0.86), p = 0.006]. However, there was no significant difference in the intensity of cells presenting TLR1, TLR4, or TLR9 between patients with AOSD and HCs. There was no significant difference in the intensity of cells presenting TLR1, TLR2, TLR4, TLR7, or TLR9 between patients with systemic and chronic articular AOSD. Furthermore, there was no significant difference in the intensity of cells presenting TLR1, TLR2, TLR4, TLR7, or TLR9 between patients with active (n = 13) and inactive AOSD (n = 7).

### The intensity of stained cells presenting TLR1, TLR2, TLR4, TLR7, and TLR9 according to the levels of disease activity markers or clinical manifestations in patients with AOSD

Correlations between the levels of disease activity markers and the intensity of stained cells presenting several TLR markers in patients with AOSD are shown in Table 2. TLR1 intensities correlated with lactate dehydrogenase (LDH) and ferritin levels. TLR2 intensities from whole cells positively correlated with systemic score and LDH and ferritin levels. TLR9 intensities also correlated with LDH and ferritin levels. However, there were no correlations between neutrophil counts and several TLR markers in patients with AOSD. TLR2 intensities from whole cells correlated with serum levels of IL-1β, IL-6, and IL-18. TLR4 intensities positively correlated with IL-1β, and TLR7 intensities correlated with IL-18.

**Table 2:** Correlation between each Toll-like receptor (TLR) level and levels of disease activity markers in adult onset Still’s disease.

Disease activity markers	Correlation coefficient, r (p-value)				
	TLR1	TLR2	TLR4	TLR7	TLR9
Systemic score	0.440 (0.052)	0.446 (0.049)	0.178 (0.452)	− 0.057 (0.811)	0.379 (0.099)
Leukocyte	− 0.200 (0.397)	− 0.261 (0.265)	− 0.342 (0.140)	− 0.265 (0.260)	− 0.286 (0.222)
Neutrophil	− 0.280 (0.232)	− 0.247 (0.295)	− 0.349 (0.132)	− 0.211 (0.373)	− 0.195 (0.409)
Hemoglobin	− 0.178 (0.453)	− 0.294 (0.208)	− 0.001 (0.996)	0.091 (0.704)	− 0.185 (0.435)
Platelet	− 0.027 (0.911)	− 0.079 (0.741)	− 0.026 (0.914)	0.140 (0.556)	− 0.180 (0.448)
ESR	− 0.058 (0.807)	− 0.013 (0.957)	− 0.194 (0.413)	− 0.194 (0.414)	− 0.107 (0.653)
CRP	− 0.015 (0.950)	− 0.052 (0.827)	− 0.138 (0.562)	− 0.172 (0.468)	− 0.056 (0.814)
Ferritin	0.804 (< 0.001)	0.829 (< 0.001)	0.547 (0.015)	0.141 (0.553)	0.798 (< 0.001)
LDH	0.716 (< 0.001)	0.728 (< 0.001)	0.465 (0.039)	0.203 (0.391)	0.686 (0.001)
Albumin	− 0.096 (0.688)	− 0.140 (0.557)	0.142 (0.549)	0.232 (0.324)	− 0.079 (0.740)
Bilirubin	0.029 (0.903)	− 0.049 (0.839)	0.038 (0.874)	0.060 (0.801)	0.056 (0.816)
AST	− 0.071 (0.765)	− 0.072 (0.762)	− 0.273 (0.244)	− 0.374 (0.104)	− 0.164 (0.491)
ALT	− 0.154 (0.516)	− 0.260 (0.269)	− 0.297 (0.204)	− 0.245 (0.298)	− 0.299 (0.200)
IL-1β	0.309 (0.071)	0.380 (0.025)	0.387 (0.022)	0.070 (0.689)	0.148 (0.395)
IL-6	0.140 (0.422)	0.474 (0.004)	0.126 (0.470)	0.267 (0.121)	0.180 (0.301)
IL-18	0.174 (0.317)	0.606 (< 0.001)	0.112 (0.523)	0.385 (0.022)	0.213 (0.219)
TNF-α	0.130 (0.457)	0.318 (0.063)	− 0.028 (0.875)	0.070 (0.689)	− 0.023 (0.895)
TLR1		0.928 (< 0.001)	0.806 (< 0.001)	0.584 (< 0.001)	0.905 (< 0.001)
TLR2	0.928 (< 0.001)		0.838 (< 0.001)	0.588 (< 0.001)	0.932 (< 0.001)
TLR4	0.806 (< 0.001)	0.838 (< 0.001)		0.669 (< 0.001)	0.813 (< 0.001)
TLR7	0.584 (< 0.001)	0.588 (< 0.001)	0.669 (< 0.001)		0.614 (< 0.001)

ESR, erythrocyte sedimentation rate; CRP, C-reactive protein; LDH, lactate dehydrogenase; AST, aspartate transaminase; ALT, alanine transaminase; IL, interleukin; TNF, tumor necrosis factor. Spearman’s correlation coefficients were calculated. The systemic scoring system of Pouchot et al.^40^ assigns a score from 0 to 12 with 1 point for each of the following manifestations: fever, typical rash, pleuritis, pneumonia, pericarditis, hepatomegaly or abnormal liver function test data, splenomegaly, lymphadenopathy, leukocytosis ≥ 15,000/mm^2^, sore throat, myalgia, and abdominal pain. These data were assessed using a Spearman’s correlation test.

We evaluated the correlation between each cell TLR marker and the remaining four TLR markers considered in this study in patients with AOSD. The intensities of each TLR marker in peripheral blood mononuclear cells (PBMCs) positively correlated with the intensities of the rest of the four TLR markers being studied (p < 0.001).

When the cell TLR marker was analysed with regard to the manifestations of AOSD, the patient who had arthritis [1.34 (0.18)] provided a higher TLR2 intensity than the patient who did not have arthritis [1.24 (0.5), p = 0.02], and the patient who had hepatosplenomegaly [1.59 (0.04)] had a higher intensity of TLR7 than those who did not have hepatosplenomegaly [1.25 (0.29), p = 0.042] (Table 3).

**Table 3:** Comparison of the intensities of Toll-like receptor2 (TLR2) and TLR7 expression according to disease manifestations in adult onset Still’s disease.

Manifestations	TLR2	p-value	TLR7	p-value
**Fever**				
(+), n = 10	1.31 ± 0.58	0.579	1.22 ± 0.23	0.353
(−), n = 10	1.23 ± 0.19		1.34 ± 0.35	
**Sore throat**				
(+), n = 4	1.58 ± 0.92	0.892	1.42 ± 0.24	0.148
(−), n = 16	1.19 ± 0.17		1.24 ± 0.30	
**Skin rash**				
(+), n = 12	1.29 ± 0.53	0.678	1.22 ± 0.22	0.305
(−), n = 8	1.24 ± 0.21		1.37 ± 0.38	
**Lymphadenopathy**				
(+), n = 3	1.68 ± 1.10	0.765	1.23 ± 0.34	0.689
(−), n = 17	1.20 ± 0.16		1.29 ± 0.30	
**Hepatosplenomegaly**				
(+), n = 2	2.10 ± 1.21	0.126	1.59 ± 0.04	0.042
(−), n = 18	1.18 ± 0.16		1.25 ± 0.29	
**Arthritis**				
(+), n = 6	1.34 ± 0.18	0.020	1.44 ± 0.41	0.239
(−), n = 12	1.24 ± 0.50		1.21 ± 0.21	

These data were assessed using a Mann–Whitney U test.

### Immunohistochemical data of the skin lesions of 32 patients with AOSD and those with eczema, psoriasis, and HC

**Figure 3:**
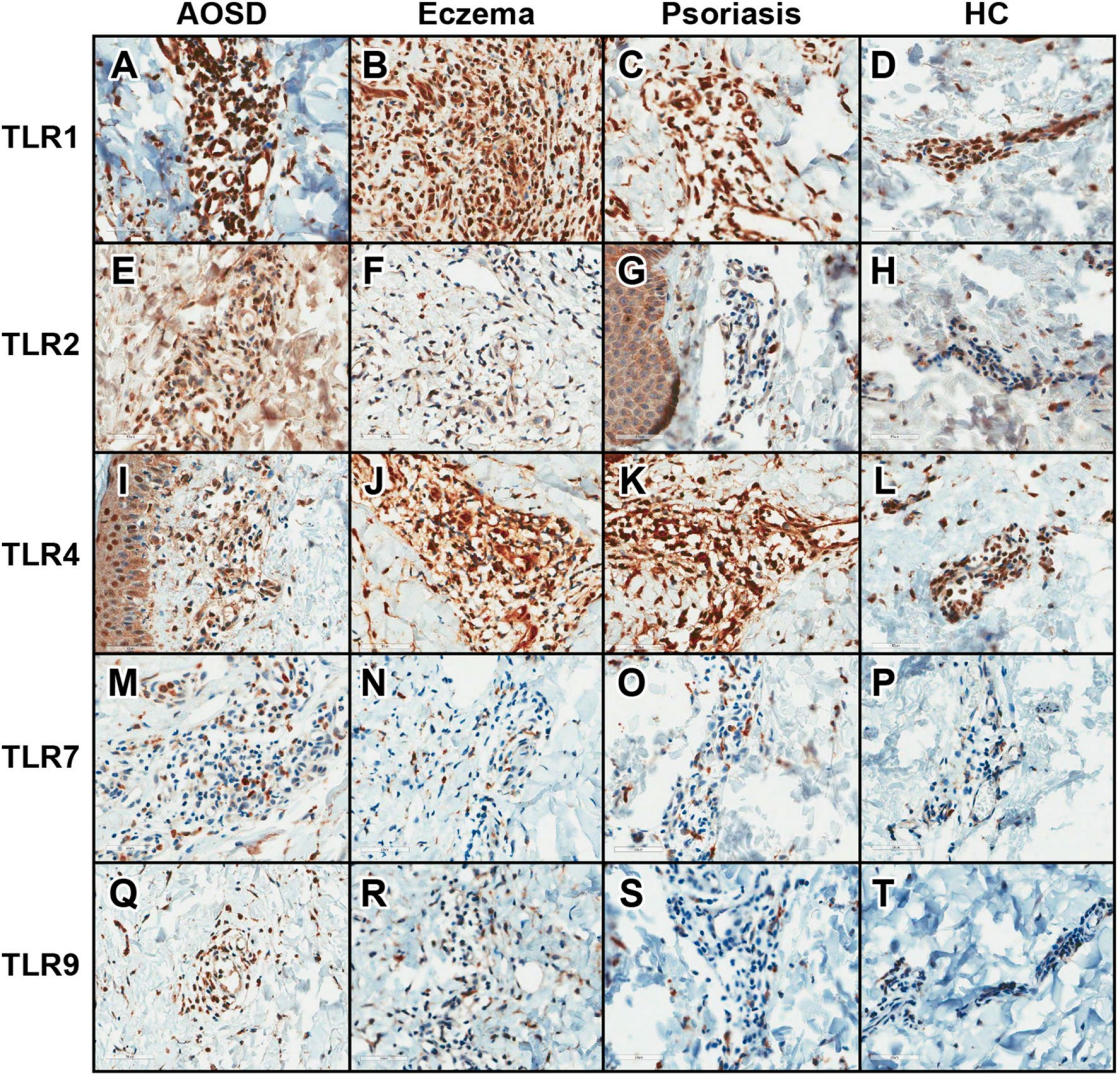
Toll-like receptor (TLR)1 (**A–D**), TLR2 (**E–H**), TLR4 (**I–L**), TLR7 (**M–P**), and TLR9 (**Q–T**) expression in skin biopsies of patients with adult-onset Still’s disease (AOSD) (**A,E,I,M,Q**), eczema (**B,F,J,N,R**), psoriasis (**C,G,K,O,S**) and healthy controls (HCs) (**D,H,L,P,T**). Original magnification, × 100 or × 400. TLR2 was more frequently expressed in the skin of patients with AOSD than in that of HCs. TLR9 was more frequently expressed in the skin of patients with AOSD than in that of patients with eczema or psoriasis, and HCs.

As a control for TLR1, TLR2, TLR4, TLR7, and TLR9 immunohistochemical (IHC) evaluation, we stained lymphoid cells in the paracortical zone or germinal centre of a reactive tonsil. This analysis revealed a granular pattern of cytoplasmic staining. The staining patterns of inflammatory cells in skin biopsies were similar to those of lymphoid cells in the tonsils. The mean percentages of inflammatory cells expressing TLR1, TLR2, TLR4, TLR7, and TLR9 in skin lesions from patients with AOSD, eczema, and psoriasis and the skin from HCs are summarized in Table 4 and Fig. 3. The comparative analysis shows that the percentage of inflammatory cells expressing TLR2 in skin lesions from patients with AOSD was significantly greater than that in HCs (p = 0.002). The percentage of inflammatory cells expressing TLR1 was greater than that of the psoriasis group (p = 0.021). Furthermore, the percentage of inflammatory cells expressing TLR9 in skin lesions from patients with AOSD was significantly greater than that in HC (p = 0.001) and patients with eczema (p = 0.041) or psoriasis (p = 0.001). We evaluated the correlations between the percentages of inflammatory cells staining for the TLRs and inflammatory cell grades or percentages, such as CD4, CD8, CD68, C-X-C motif chemokine 9 (CXCL9), CXCL10, CXCL11, CXCL12, C-X-C chemokine receptor type 3 (CXCR3), and CXCR4 (Table S2). The expression of TLR2 correlated significantly with CXCL10-stained inflammatory cells in the skin of patients with AOSD. The expression of TLR4 correlated significantly with inflammatory cells stained for CD4, CXCL11, and CXCR3. TLR9 staining positively correlated with inflammatory cells stained for CXCL11 and CXCL12.

**Table 4:** Toll-like receptor (TLR) immunostaining results in skin biopsies from patients with adult-onset Still’ disease (AOSD), eczema, and psoriasis and healthy controls (HC).

	Staining cell percent AOSD (n = 32)	Staining cell percent eczema (n = 5)	p-value (AOSD vs. eczema)	Staining cell percent psoriasis (n = 5)	p-value (AOSD vs. psoriasis)	Staining cell percent HC (n = 5)	p-value (AOSD vs. HC)
TLR1	81.9 ± 8.2	70.0 ± 16.0	0.097	72.0 ± 5.7	0.021	70.0 ± 18.4	0.128
TLR2	34.8 ± 24.1	27.0 ± 30.9	0.328	27.2 ± 21.4	0.620	6.2 ± 3.6	0.002
TLR4	52.3 ± 29.8	75.0 ± 10.0	0.117	64.0 ± 11.4	0.620	77.0 ± 11.0	0.071
TLR7	19.3 ± 15.7	24.4 ± 31.2	0.682	11.0 ± 4.2	0.213	9.6 ± 6.6	0.106
TLR9	40.2 ± 27.9	17.6 ± 20.2	0.041	7.4 ± 9.9	0.001	5.4 ± 4.5	0.001

p-values determined using a Mann–Whitney U-test. AOSD, adult-onset Still’s disease; HC, healthy control.

### Immunohistochemical data for LNs

**Figure 4:**
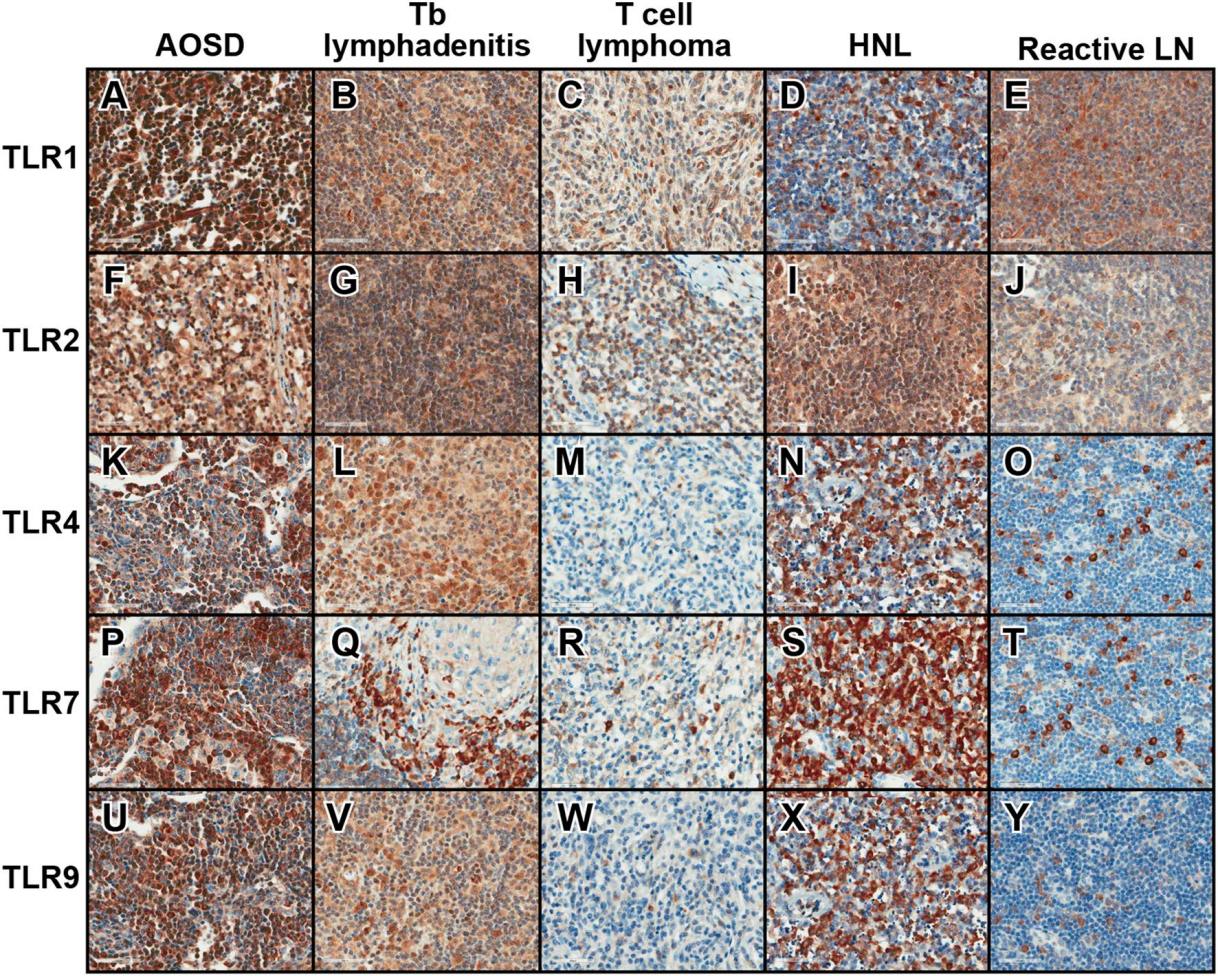
Toll-like receptor (TLR)1 (**A-E**), TLR2 (**F-J**), TLR4 (**K-O**), TLR7 (**P-T**), and TLR9 (**U-Y**) expression in lymph node (LN) biopsies of patients with adult-onset Still’s disease (AOSD) (**A,F,K,P,U**), tuberculosis (Tb) lymphadenitis (**B,G,L,Q,V**), T cell lymphoma (**C,H,M,R,W**), histiocytic necrotizing lymphadenitis (HNL) (**D,I,N,S,X**), and reactive lymphadenopathy (**E,J,O,T,Y**). Original magnification, × 100 or × 400. TLR1 was more frequently expressed in the LN of patients with AOSD than in that of patients with Tb lymphadenitis, T cell lymphoma or reactive LN. TLR2 was more frequently expressed in the LN of patients with AOSD than in that of patients with HNL. TLR4 was more frequently expressed in the LN of patients with AOSD than in that of patients with T cell lymphoma or reactive LN. TLR7 ware more frequently expressed in the LN of patients with AOSD than in that of patients with T cell lymphoma or reactive LN. TLR9 was more frequently expressed in the LN of patients with AOSD than in that of patients with T cell lymphoma or reactive LN.

The IHC findings for TLR1, TLR2, TLR4, TLR7, and TLR9 of LNs are shown in Table 5 and Fig. 4. The table summarizes the mean percentages of inflammatory cells expressing TLR1, TLR2, TLR4, TLR7, and TLR9 in LNs from patients with AOSD, tuberculosis (Tb) lymphadenitis, T cell lymphoma, histiocytic necrotizing lymphadenitis (HNL), and reactive LNs. The comparative analysis shows that the percentage of inflammatory cells expressing TLR1 in LNs from patients with AOSD was significantly greater than that in patients with TB lymphadenitis (p = 0.029), T cell lymphoma (p = 0.004), and reactive LN (p = 0.004). The percentage of inflammatory cells expressing TLR2 was greater than that in HNL (p = 0.042). Furthermore, the percentage of inflammatory cells expressing TLR4, TLR7, and TLR9 in LNs from patients with AOSD was significantly greater than that in patients with T cell lymphoma (p = 0.001) and reactive LNs (p = 0.012).

**Table 5:** Toll-like receptor (TLR) immunostaining results in lymph node (LN) biopsies from patients with adult-onset Still’ disease (AOSD) and other similar conditions.

	Staining cell percent AOSD (n = 9)	Staining cell percent Tb lymphadenitis (n = 5)	p-value (AOSD vs. Tb lymphadenitis)	Staining cell percent T cell lymphoma (n = 5)	p-value (AOSD vs. T cell lymphoma)	Staining cell percent HNL (n = 5)	p-value (AOSD vs. HNL)	Staining cell percent reactive LN (n = 5)	p-value (AOSD vs. reactive LN)
TLR1	34.4 ± 17.9	13.2 ± 13.2	0.029	9.2 ± 6.2	0.004	24.0 ± 2.9	0.240	7.6 ± 7.6	0.004
TLR2	17.3 ± 16.6	6.8 ± 5.7	0.190	20.0 ± 31.8	0.438	4.8 ± 3.0	0.042	8.6 ± 4.7	0.518
TLR4	31.1 ± 13.6	44.0 ± 26.0	0.364	4.4 ± 3.4	0.001	52.0 ± 17.5	0.060	12.2 ± 7.2	0.012
TLR7	27.2 ± 20.0	32.0 ± 4.5	0.190	8.2 ± 7.2	0.019	49.0 ± 12.5	0.060	8.0 ± 2.7	0.002
TLR9	18.3 ± 9.7	11.2 ± 11.2	0.147	3.6 ± 4.0	0.002	21.0 ± 19.2	0.898	2.6 ± 1.7	0.001

p-values determined using a Mann–Whitney U-test. AOSD, adult-onset Still’s disease; Tb, tuberculosis; HNL, histiocytic necrotizing lymphadenitis; LN, lymph node.

## Discussion

Patients with AOSD showed significantly higher intensities of cells presenting TLR2 and TLR7 from whole blood than HCs. In particular, the TLR2 intensities from whole blood cells correlated with serum inflammatory cytokine levels and several disease activity markers, such as systemic score, LDH, and ferritin. Furthermore, the intensity of each TLR correlated with that of other TLRs. The expression of TLR2-positive inflammatory cells was higher in skin biopsy samples from patients with AOSD than in those from HCs. TLR9-expressing positive inflammatory cell counts were higher in skin lesions from patients with AOSD than in HC, eczema, and psoriasis groups. The expression levels of TLR1, TLR4, TLR7, and TLR9 were higher in LNs of patients with AOSD than in LNs of those with T cell lymphoma and reactive lymphadenopathy.

The role of the innate immune system has been investigated in the initiation and progression of several autoimmune or inflammatory diseases. In particular, TLR-mediated inflammation is implicated in several rheumatic diseases, including rheumatoid arthritis (RA), systemic lupus erythematosus (SLE), and systemic sclerosis. TLR2, TLR3, TLR4, and TLR7 were overexpressed in the macrophages of synovial tissues, and the levels of TLR2 and TLR4 expression were correlated with IL-12 and IL-18 levels in the synovial tissue of patients with RA^24, 25^. TLR7 was highly expressed in mouse models of severe lupus and in patients with SLE, and inhibition of TLR7 signalling ameliorated inflammation of the lungs and kidneys in lupus-prone mice^26, 27^. Several TLR ligands as DAMPs were evaluated in the serum of patients with active AOSD; serum S100A8/A9, S100A12, HMGB1, and amyloid A levels increased in patients with active AOSD^2, 15, 16, 20, 22^. However, few studies have evaluated TLR in the PBMCs of patients with AOSD. Only one study evaluated the expression of TLR7 on circulating pre-mDCs and mDCs in patients with AOSD^23^. The transcript levels of TLR7 and associated signalling molecules, including Myd88, TRAF6, and IFN-α, were correlated with the serum cytokine levels of IL-1β and IFN-α. The levels of NLRP3 inflammasomes and their by-products increased in patients with AOSD and correlated with disease activity^28^. Interestingly, the levels of NLRP3 inflammasome pathway components were upregulated by a TLR7 agonist in PBMCs of patients with AOSD but not in those of HCs. Our study showed that the TLR2 and TLR7 intensities of PBMCs in patients with AOSD were significantly higher than those in HCs. These results could be interpreted in the same context as previous data related to TLR7 in AOSD^23^. Furthermore, TLR2 intensities correlated with the levels of several inflammatory cytokines and disease activity markers and systemic scores and were higher in patients with arthritis than in those without arthritis. These results strongly suggest that the expression of TLR2 on circulating mononuclear cells may play a significant role in the pathogenesis, clinical manifestation, and disease activity of AOSD. TLR2 is an extracellular receptor, and its signalling via MyD88 is triggered by endogenous DAMPs, such as heat shock protein, serum amyloid A, HMGB1, and β-defensin 3^14, 20^. The levels of these endogenous DAMPs are already elevated in the serum of patients with AOSD or systemic juvenile idiopathic arthritis^15, 29^; they could interact with TLR2 in circulating mononuclear cells. These interactions can aggravate inflammation in the pathogenesis of AOSD. Furthermore, Dectin-1 was found to physically interact and synergize with TLR2 to induce IL-1β and TNF-α in human PBMCs^30^. A recent study evaluated the frequency of blood cells expressing Dectin-1 between AOSD and HC and showed that the frequency of granulocytes presenting Dectin-1 was increased in the active phase of AOSD compared to that in the inactive phase^31^. However, our microarray results showed the expression levels of TLR1, TLR2, and TLR4, not TLR7 were increased in active AOSD compared to HCs. These discrepancies between microarray and FACS data could be explained by the difference between RNA and protein expression levels, and the difference enrolled patients characteristics.

We analysed the expression of several TLRs during the initial phase of AOSD, using skin and LN biopsy specimens obtained before treatment. The skin of patients with AOSD showed increased expression of TLR2 and TLR9 compared to that in HCs. In particular, increased expression of TLR9 was observed in the skin lesions of patients with AOSD compared to that of HCs, eczema, and psoriasis groups. The expression of TLR2 correlated significantly with CXCL10-stained inflammatory cells in the skin of patients with AOSD. TLR9 staining positively correlated with inflammatory cells stained for CXCL11 and CXCL12. These results suggest that TLR2 and TLR9 induce chemokine production during skin inflammation in AOSD. TLR2 expression and some skin or inflammatory diseases, such as atopic dermatitis and erythema nodosum reprosum, were shown^32–34^. One study showed that TLR2-mediated sensing of *Staphylococcus aureus*-derived signalling was strongly impaired in Langerhans cells from atopic dermatitis skin^33^. On the other way, the other study suggested that innate TLR2 signals convert transient T helper 2 cell-mediated dermatitis into persistent inflammation, as seen in chronic atopic dermatitis, through IL-4-mediated suppression of IL-10^35^. TLR9 is well known as a receptor that responds to nucleic acids, and there have been several reports that showed association with skin lesions of autoimmune diseases^36, 37^. A recent study suggested that systemic sclerosis immune complexes have the potential pathogenicity mediated by TLR9 via the interaction with nucleic acid fragments on fibroblasts^38^. Interestingly, highly expressed TLR in the lymphadenopathy of AOSD differed based on the specific lymphadenopathy. The expression levels of TLR1, TLR4, TLR7, and TLR9 increased in the LNs of patients with AOSD compared to that in reactive LN or T cell lymphoma. However, TLR2 expression increased in the LNs of patients with AOSD compared to that in HNL. Furthermore, the expression level of TLR1 was higher in the LNs of patients with AOSD than in Tb lymphadenopathy. One study analysed the expression of TLR1 to 9 using quantitative real-time PCR in frozen LN samples from patients with follicular lymphoma, diffuse large B-cell lymphoma, and peripheral T-cell lymphoma^39^. TLR expression was highly variable among lymphoma subtypes; for example, TLR5 showed lower expression in follicular lymphoma, and TLR2 was overexpressed in both diffuse large B-cell lymphoma and peripheral T-cell lymphoma. In this study, we recruited LNs from patients with T-cell lymphoma for differential diagnosis of LNs in AOSD. The expression level of TLR2 was similar in LNs from patients with T cell lymphoma and AOSD; however, TLR1, TLR4, TLR7, and TLR9 expression levels were higher in the LNs of patients with AOSD than in T cell lymphoma. Therefore, immunohistochemical analysis of TLR expression in the LNs could be an additional marker for differentiating several diseases with similar clinical manifestations.

This study had some limitations. TLR markers were not compared with those of other febrile diseases as positive controls in terms of their diagnostic value, and the sample sizes were small for comparison of TLR expression between systemic and chronic articular manifestations of AOSD. There were no follow-up blood samples or skin or LN biopsies in AOSD for the comparison of TLR expression. The disease activities of the AOSD were relatively low with systemic score 2.5 ± 1.9, and could affect relatively low IL-1β and TNF-α levels in serum of the AOSD patients. However, we recruited the patients prospectively, observed several TLRs in AOSD and evaluated their roles in assessing disease activity and expression in the skin and LNs of patients with AOSD and compared their expression with TLR expression of several similar diseases. Based on this study, it is necessary to determine whether the symptoms of AOSD could be improved by regulating the TLR sub-signalling system in the future. Further studies including larger sample sizes with follow-up blood samples and control groups including other febrile disorders are required to assess the usefulness of TLR expression in patients with AOSD. Additionally, identifying other DAMPs and PAMPs that could stimulate TLR expression and TLR expressed cellular types and characteristics in AOSD would be helpful in understanding the pathogenesis and treatment of AOSD. This study targeted only TLR1, TLR2, TLR4, TLR7 and TLR9. Further studies are needed for the other TLRs in AOSD.

In conclusion, we found significantly higher numbers of circulating TLR2-positive cells in patients with AOSD. Furthermore, numbers of circulating TLR2-positive cells were increased in patients with AOSD and arthritis compared to that in patients without arthritis. We confirmed the expression of these PRRs in skin rash tissue from patients with AOSD. These results suggest that TLR2 may play an important role in the systemic inflammatory process and arthritis in AOSD. Furthermore, immunohistochemical staining for TLRs in skin lesions and LNs may facilitate differentiating AOSD from other similar conditions.

## Materials and methods

### Subjects

A total of 20 patients with AOSD and 15 HCs were included in the study. Patients with AOSD were diagnosed according to Yamaguchi’s criteria; patients with infections, other autoimmune diseases, and malignancies were excluded. The HCs were individuals with no history of autoimmune, rheumatic, or other malignant diseases. PBMCs were isolated from the study participants. Fifteen of the 20 patients with AOSD with systemic inflammation were enrolled in the study. Among them, nine were in the initial stages of high-level disease activity before treatment. Six patients experienced a systemic flare-up of the disease during follow-up. The remaining five patients had chronic articular pattern disease.

The medical histories and clinical characteristics, including those identified during the physical examination of all subjects, were collected after reviewing the subjects’ medical records and interviewing the subjects during sample collection. The complete blood count, erythrocyte sedimentation rate, C-reactive protein and ferritin levels, and liver function test results were reviewed. The disease activity of AOSD was assessed according to the widely accepted systemic scoring method^40^. The inactive disease was defined as the absence of systemic symptoms such as fever, myalgia, skin rash, and pericarditis. This study was approved by the Institutional Review Board (IRB) of Ajou University Hospital (IRB No. AJIRB-BMR-OBS-19-053), and was conducted in compliance with the principles of the Declaration of Helsinki. Informed consent was obtained from all subjects.

### Microarray data analysis

PBMCs of 2 active AOSD patients, 2 inactive AOSD patients, and 2 HCs were included in microarray data analysis. CEL files were imported into the Gene Expression Workflow in GeneSpring GX version 14.9.1 (Agilent Technologies Inc.). The RMA algorithm (background correction, log2 transformation, and probeset summarization) was performed using the default settings in the GeneSpring software. The principal component analysis, which reduces the dimensionality of a dataset consisting of a large number of interrelated variables, was performed using a covariance dispersion matrix as part of the quality control of the data. Differential expression (DE) between exposed (treated) and unexposed (control) rats was predicted at the gene level (probesets summarized into transcript clusters/genes). Unpaired *t*-test was used to compare the individual gene expression data with respect to treated versus control groups. DE genes were defined based on an absolute fold change equal to or greater than 2.0 and a p-value ≤ 0.05. The input for the heatmap (TreeView Ver.1.1.6r4) was the log2-transformed intensities of DE genes with an absolute fold change equal to or greater than 2.0 and a p-value ≥ 0.05.

### Flow cytometry of cells presenting TLR1, TLR2, TLR4, TLR7, and TLR9

Twenty patients with AOSD and 15 HC subjects were used for fluorescence-activated cell sorting (FACS) analysis. PBMCs were isolated using CPT™ Mononuclear Cell preparation tube-BD Vacutainer® (BD Biosciences, Franklin Lakes, NJ, USA). For anti-TLR7 and TLR9 intracellular antibody reactions, cells were pre-treated with Tween 20. Cells (1 × 10^6^) in each tube were incubated with FACS blocking buffer (3% BSA in PBS) at 4 °C for 1 h. After blocking, fluorescein isothiocyanate (FITC)-labelled anti-TLR1 (Abcam, Cambridge, MA, USA), anti-TLR2 (Abcam), anti-TLR7 (Thermo Fisher Scientific Inc., Waltham, MA, USA), and anti-TLR9 (Abcam) and phycoerythrin-labelled anti-TLR4 (Abcam) were added and incubated for 1 h at 4 °C. The same colour-labelled antibodies were applied separately to different tubes for isotype control. The stained cells were then washed with FACS buffer and analysed for 10,000 cells using a flow cytometer (FACSAria III; Becton, Dickinson and Company, San Jose, CA, USA). The FACS data were based on specific markers and were used to analyse the gated populations. The density was plotted using FlowJo V.10 software (Becton, Dickinson and Company, Ashland, OR, USA).

### Cytokine assay

Venous blood of the AOSD patients and HCs was extracted in serum separator tubes, containing an anticoagulant, EDTA, and aliquots of the blood were centrifuged for 10 min at 2500 rpm. After aspirating the serum, the samples were stored at − 20 °C. Serum IL-1β, IL-6, IL-18 and TNFα levels were measured using enzyme-linked immunosorbent assay kits (R&D Systems, Inc., Minneapolis, MN) according to the manufacturer’s protocol.

### Histopathological evaluation of skin and lymph node specimens

Skin biopsy materials were retrospectively collected from 32 patients with active AOSD, between 2000 and 2019, for the evaluation of skin rashes. Informed consent was waived by the IRB because of the retrospective nature of the study. The haematoxylin and eosin (H&E)-stained sections were independently reviewed by three pathologists to evaluate epidermal changes, including epidermal necrosis, vacuolization of basal keratinocytes, parakeratosis, the presence of macrophage infiltration, inflammatory cell infiltration, and karyorrhexis. We examined the H&E-stained sections of excisional LN biopsy samples obtained from nine patients with AOSD.

### Immunohistochemical staining for TLRs in 32 skin and nine LN samples from patients with active AOSD

Formalin-fixed, paraffin-embedded sections were analysed by immunohistochemistry using a Benchmark XT automated staining system (Ventana Medical Systems Inc., Tucson, AZ, USA). The primary antibodies used were anti-TLR1 (1:500; Abcam, Cambridge, MA), TLR2 (1:100 for LNs and 1:200 for skin; Abcam), TLR4 (1:400; Santa Cruz Biotechnology, Santa Cruz, CA, USA), TLR7 (1:100; Abcam), and TLR9 (1:400; Abcam). Staining was observed using the Ventana Optiview DAB kit (Ventana Medical Systems Inc., Oro Valley, AZ, USA). The IHC results for TLR1, TLR2, TLR4, TLR7, and TLR9 were graded according to the percentage of positive lymphoid cells and histiocytes.

### Statistical analyses

Continuous variables were expressed as mean (standard deviation), and categorical variables were expressed as frequencies with percentages. The Mann–Whitney U test was used to compare TLR expression levels in PBMCs from patients with AOSD and HCs and in the skin and LNs from patients with AOSD and those with control diseases. We calculated Spearman correlations between TLR expression levels and systemic disease activity levels. All statistical analyses were performed using SPSS (version 23.0; SPSS, Chicago, IL, USA). p values < 0.05 indicated statistical significance.

### Ethics declarations and approval for human experiments

The study was approved by the institutional review board of Ajou University Hospital (IRB No. AJIRB-BMR-OBS-19-053).

### Consent to participate and consent to publish

All study participants provided informed consent.

## Supplementary Information

Supplementary Information.

## Data Availability

All available data are reported in the manuscript and supplementary file.
