# Publisher Correction: Elevated expression of TLR2 and its correlation with disease activity and clinical manifestations in adult-onset Still’s disease

**DOI:** 10.1038/s41598-022-16346-5

**Published:** 2022-07-13

**Authors:** Jae Ho Han, Mi-Hyun Ahn, Ju-Yang Jung, Ji-Won Kim, Chang-Hee Suh, Ji Eun Kwon, Hyunee Yim, Hyoun-Ah Kim

**Affiliations:** 1 Department of Pathology, Ajou University School of Medicine, Suwon, Korea; 2 Department of Rheumatology, Ajou University School of Medicine, 164 Worldcup-ro, Yeongtong-gu, Suwon, 16499 Korea

Correction to: *Scientific Reports* https://doi.org/10.1038/s41598-022-14004-4, published online 17 June 2022

In the original version of this Article, Hyoun-Ah Kim was incorrectly affiliated with ‘Department of Pathology, Ajou University School of Medicine, Suwon, Korea’. The correct affiliation is listed below.

Department of Rheumatology, Ajou University School of Medicine, 164 Worldcup-ro, Yeongtong-gu, Suwon, 16,499, Korea

Additionally, Hyoun-Ah Kim was erroneously listed as equally contributing author. Equal contribution statement was correct in the PDF version of the Article at the time of publication.

The original Article has been corrected.

