# Supplementary material for: Elevated expression of TLR2 and its correlation with disease activity and clinical manifestations in adult-onset Still’s disease
Source: Sci Rep. 2022 Jun 17;12:10240. doi: 10.1038/s41598-022-14004-4 (PMC9205149; doi:10.1038/s41598-022-14004-4)
Supplement: Supplementary file 1 — Supplementary Information. [file 41598_2022_14004_MOESM1_ESM.docx]

Supplementary Table S1. Total expression profiles of selected gene list.

| **GenbankAccessionID** | **UniGeneID** | **active AOSD vs. HC** | **FCAbsolute[active AOSD vs. HC]** | **p-val[active AOSD vs. HC]** | **Regulation[active AOSD vs. HC]** | **improved AOSD vs. HC** | **FCAbsolute[improved AOSD vs. HC]** | **p-val[improved AOSD vs. HC]** | **Regulation[improved AOSD vs. HC]** | **active AOSD vs. improved AOSD** | **FCAbsolute[active AOSD vs. improved AOSD]** | **p-val[active AOSD vs. improved AOSD]** | **Regulation[active AOSD vs. improved AOSD]** |
| --- | --- | --- | --- | --- | --- | --- | --- | --- | --- | --- | --- | --- | --- |
| NM_006908 | Hs.413812 | 0.01250667 | 1.00870664 | 0.9170616 | up | -0.02008333 | 1.01401805 | 0.8673038 | down | 0.03259 | 1.02284675 | 0.8048211 | up |
| NM_006908 | Hs.413812 | 0.0525 | 1.03706046 | 0.7960943 | up | 0.146945 | 1.10722237 | 0.4789031 | up | -0.094445 | 1.06765461 | 0.6729782 | down |
| NM_006908 | Hs.413812 | 0.12210167 | 1.08831913 | 0.4751143 | up | -0.33646333 | 1.26265748 | 0.08308814 | down | 0.458565 | 1.37417429 | 0.0422924 | up |
| NM_014294 | Hs.491988 | -0.41174833 | 1.33029696 | 0.06955371 | down | 0.27042667 | 1.20616449 | 0.1948728 | up | -0.682175 | 1.60455695 | 0.01646868 | down |
| NM_014294 | Hs.491988 | -0.86982833 | 1.82744544 | 0.0315681 | down | -0.62559833 | 1.54285056 | 0.08952799 | down | -0.24423 | 1.18446043 | 0.4938559 | down |
| NM_001014431 | Hs.525622 | -0.75937 | 1.69275127 | 0.00079798 | down | 0.79998 | 1.74107699 | 0.000614 | up | **-1.55935** | 2.94721028 | 2.99E-05 | down |
| NM_003824 | Hs.86131 | 0.05528167 | 1.03906195 | 0.7656916 | up | 0.32048667 | 1.24875172 | 0.1234597 | up | -0.265205 | 1.20180681 | 0.2232583 | down |
| NM_003824 | Hs.86131 | 0.24169667 | 1.18238237 | 0.3443044 | up | 0.08235667 | 1.05874611 | 0.7377181 | up | 0.15934 | 1.11677612 | 0.558643 | up |
| NM_012288 | Hs.520182 | -0.08077333 | 1.05758479 | 0.6029587 | down | -0.12846333 | 1.09312875 | 0.4170035 | down | 0.04769 | 1.03360862 | 0.7772068 | up |
| NM_012288 | Hs.520182 | 0.22380333 | 1.16780819 | 0.357103 | up | -0.16399167 | 1.12038274 | 0.4918154 | down | 0.387795 | 1.30839214 | 0.1673128 | up |
| NM_012288 | Hs.520182 | -0.49596333 | 1.41026212 | 0.03568578 | down | 0.30946167 | 1.2392452 | 0.1399646 | up | -0.805425 | 1.74766056 | 0.00757912 | down |
| NM_001165412 | Hs.654408 | 0.36105833 | 1.28436774 | 0.02652226 | up | 0.49204833 | 1.40644032 | 0.00762711 | up | -0.13099 | 1.09504488 | 0.3622636 | down |
| NM_013254 | Hs.505874 | 0.36279833 | 1.28591772 | 0.5013172 | up | -0.76077667 | 1.69440255 | 0.1863043 | down | 1.123575 | 2.17886226 | 0.09216828 | up |
| NM_013254 | Hs.505874 | 0.43787167 | 1.35460447 | 0.4315812 | up | 0.33806667 | 1.26406151 | 0.5389104 | up | 0.099805 | 1.07162861 | 0.866237 | up |
| NM_001197122 | Hs.75254 | 0.25006667 | 1.18926207 | 0.5139548 | up | 0.43993167 | 1.35654007 | 0.2696173 | up | -0.189865 | 1.14065697 | 0.6475205 | down |
| NM_001572 | Hs.166120 | **1.8958983** | 3.72153637 | 0.01934037 | up | 1.03335833 | 2.04678325 | 0.1301408 | up | 0.86254 | 1.81823667 | 0.2285922 | up |
| NM_006116 | Hs.507681 | -0.03690667 | 1.02591177 | 0.8799232 | down | 0.06117333 | 1.04331394 | 0.8026541 | up | -0.09808 | 1.07034805 | 0.7154631 | down |
| NM_006116 | Hs.507681 | 0.01529833 | 1.01066042 | 0.9738088 | up | -0.21734667 | 1.16259343 | 0.6443217 | down | 0.232645 | 1.17498717 | 0.6517646 | up |
| NM_001172566 | Hs.82116 | 0.19768333 | 1.14685527 | 0.351285 | up | 0.68738833 | 1.61036568 | 0.01394492 | up | -0.489705 | 1.40415773 | 0.06454124 | down |
| NM_001172566 | Hs.82116 | **1.1312217** | 2.19044147 | 0.01937175 | up | 0.87207167 | 1.83028925 | 0.04906005 | up | 0.25915 | 1.19677339 | 0.5232818 | up |
| NM_001014431 | Hs.525622 | 0.30452333 | 1.23501052 | 0.1345971 | up | 0.52947833 | 1.44340718 | 0.02503929 | up | -0.224955 | 1.16874079 | 0.2859384 | down |
| NM_001014431 | Hs.525622 | -0.324575 | 1.25229547 | 0.0453435 | down | -0.28864 | 1.22148826 | 0.06574789 | down | -0.035935 | 1.02522105 | 0.8043451 | down |
| NM_001077493 | Hs.73090 | -0.06294833 | 1.04459835 | 0.7818391 | down | 0.08473167 | 1.06049048 | 0.7101598 | up | -0.14768 | 1.10778661 | 0.55826 | down |
| NM_006908 | Hs.413812 | -0.00291833 | 1.00202488 | 0.9914082 | down | 0.07537167 | 1.05363244 | 0.7817093 | up | -0.07829 | 1.05576592 | 0.7926729 | down |
| NM_006908 | Hs.413812 | 0.32468333 | 1.25238951 | 0.733951 | up | 2.15776333 | 4.46222522 | 0.05847509 | up | -1.83308 | 3.56296917 | 0.1189705 | down |
| NM_001098627 | Hs.521181 | 0.00870333 | 1.00605092 | 0.946255 | up | -0.04099667 | 1.02882433 | 0.7519512 | down | 0.0497 | 1.03504967 | 0.7267854 | up |
| NM_001098627 | Hs.521181 | 0.07354833 | 1.05230166 | 0.6525624 | up | 0.11228333 | 1.08093767 | 0.4973341 | up | -0.038735 | 1.02721274 | 0.8273631 | down |
| NM_001098627 | Hs.521181 | 0.26312667 | 1.20007674 | 0.7359049 | up | 0.53647167 | 1.45042095 | 0.4989042 | up | -0.273345 | 1.20860683 | 0.7489476 | down |
| NM_001080124 | Hs.599762 | 0.62622333 | 1.5435191 | 0.04671476 | up | **1.2493233** | 2.37729894 | 0.00283147 | up | -0.6231 | 1.5401811 | 0.06336759 | down |
| NM_001626 | Hs.631535 | -0.040765 | 1.02865914 | 0.8113297 | down | 0.234915 | 1.1768374 | 0.2030703 | up | -0.27568 | 1.21056454 | 0.1771242 | down |
| NM_001626 | Hs.631535 | -0.17123833 | 1.12602459 | 0.4089254 | down | 0.12540167 | 1.09081139 | 0.5392879 | up | -0.29664 | 1.22828045 | 0.2114415 | down |
| NM_001626 | Hs.631535 | 0.09528 | 1.06827272 | 0.3551524 | up | 0 | 1 | 1 | down | 0.09528 | 1.06827272 | 0.395511 | up |
| NM_006116 | Hs.507681 | 0.25685333 | 1.19486973 | 0.2263249 | up | 0.16154333 | 1.118483 | 0.4275844 | up | 0.09531 | 1.06829493 | 0.6624193 | up |
| NM_001025242 | Hs.522819 | 0.30082833 | 1.23185149 | 0.1069233 | up | 0.57894833 | 1.49375996 | 0.0116169 | up | -0.27812 | 1.21261368 | 0.1599335 | down |
| NM_001114182 | Hs.138499 | -0.12921333 | 1.09369717 | 0.3080327 | down | 0.13867167 | 1.10089103 | 0.277353 | up | -0.267885 | 1.2040414 | 0.0816206 | down |
| NM_001114182 | Hs.138499 | 0.33289333 | 1.25953685 | 0.1884103 | up | -0.21507667 | 1.1607656 | 0.3730547 | down | 0.54797 | 1.46202705 | 0.06875067 | up |
| NM_001039661 | Hs.537126 | -0.14159333 | 1.10312275 | 0.3735709 | down | 0.04078667 | 1.02867459 | 0.7905881 | up | -0.18238 | 1.13475434 | 0.3020233 | down |
| NM_001039661 | Hs.537126 | 0.125855 | 1.0911542 | 0.4092739 | up | 0.037975 | 1.02667175 | 0.7974869 | up | 0.08788 | 1.06280727 | 0.5917837 | up |
| NM_005465 | Hs.498292 | -0.03035167 | 1.02126103 | 0.7772649 | down | -0.06294667 | 1.04459715 | 0.5623115 | down | 0.032595 | 1.02285029 | 0.7814991 | up |
| NM_005465 | Hs.498292 | -0.14133167 | 1.10292269 | 0.2644543 | down | -0.07088667 | 1.05036203 | 0.5583215 | down | -0.070445 | 1.05004052 | 0.5943506 | down |
| NM_005465 | Hs.498292 | 0.10779833 | 1.0775825 | 0.4900093 | up | -0.15204167 | 1.11114082 | 0.34057 | down | 0.25984 | 1.19734591 | 0.1590671 | up |
| NM_001114182 | Hs.138499 | 0.11366 | 1.08196963 | 0.486121 | up | -0.00304 | 1.00210939 | 0.9847816 | down | 0.1167 | 1.08425192 | 0.5127069 | up |
| NM_001080124 | Hs.599762 | 0.403865 | 1.32304763 | 0.1935354 | up | **1.54913** | 2.92640613 | 0.00167772 | up | **-1.145265** | 2.21186755 | 0.01005331 | down |
| NM_001114182 | Hs.138499 | 0.504115 | 1.41825308 | 0.2114078 | up | -0.13586 | 1.09874759 | 0.7176421 | down | 0.639975 | 1.55830216 | 0.1568062 | up |
| NM_001080124 | Hs.599762 | -0.273005 | 1.20832203 | 0.1763891 | down | 0.25287 | 1.1915752 | 0.2053031 | up | -0.525875 | 1.43980656 | 0.03733173 | down |
| NM_015093 | Hs.269775 | 0.37172333 | 1.2938975 | 0.1484794 | up | 0.99295333 | 1.99025506 | 0.00506165 | up | -0.62123 | 1.53818604 | 0.04606225 | down |
| NM_004620 | Hs.591983 | 0.030385 | 1.02128463 | 0.7276503 | up | 0.02735 | 1.01913841 | 0.7536609 | up | 0.003035 | 1.00210592 | 0.9745201 | up |
| NM_001197122 | Hs.75254 | 0.40857667 | 1.32737561 | 0.1643093 | up | 0.82024167 | 1.76570174 | 0.02033367 | up | -0.411665 | 1.33022012 | 0.1950106 | down |
| NM_001197122 | Hs.75254 | 0.05938333 | 1.04202026 | 0.846722 | up | 0.36724833 | 1.28989026 | 0.2605581 | up | -0.307865 | 1.23787445 | 0.3776729 | down |
| NM_001626 | Hs.631535 | 0.067975 | 1.04824431 | 0.7271979 | up | 0.95091 | 1.9330916 | 0.00270213 | up | -0.882935 | 1.84412315 | 0.00576724 | down |
| NM_001626 | Hs.631535 | 0.11723 | 1.08465031 | 0.3643677 | up | 0.155835 | 1.11406623 | 0.240845 | up | -0.038605 | 1.02712018 | 0.7773212 | down |
| NM_001098627 | Hs.521181 | -0.17393667 | 1.12813261 | 0.2050679 | down | 0.18823333 | 1.13936764 | 0.1753045 | up | -0.36217 | 1.28535779 | 0.03709516 | down |
| NM_001172566 | Hs.82116 | 0.14705 | 1.10730296 | 0.5598251 | up | **1.300855** | 2.46374851 | 0.00197354 | up | **-1.153805** | 2.22499948 | 0.00526719 | down |
| NM_001172566 | Hs.82116 | -0.00575333 | 1.00399587 | 0.9806356 | down | 0.95125167 | 1.93354946 | 0.00676715 | up | -0.957005 | 1.94127567 | 0.00976766 | down |
| NM_001626 | Hs.631535 | -0.042095 | 1.02960788 | 0.694374 | down | -0.08363 | 1.05968098 | 0.4446526 | down | 0.041535 | 1.0292083 | 0.7230279 | up |
| NM_006116 | Hs.507681 | 0.19526333 | 1.14493312 | 0.4160378 | up | 0.45387833 | 1.36971746 | 0.09097878 | up | -0.258615 | 1.19632967 | 0.3321396 | down |
| NM_001626 | Hs.631535 | -0.03641833 | 1.02556457 | 0.9026943 | down | 0.01963667 | 1.01370415 | 0.9474134 | up | -0.056055 | 1.03961907 | 0.8637025 | down |
| NM_001080124 | Hs.599762 | 0.99926667 | 1.99898364 | 0.01144956 | up | 0.88991167 | 1.85306266 | 0.01827539 | up | 0.109355 | 1.07874584 | 0.724551 | up |
| NM_015093 | Hs.269775 | 0.13840333 | 1.10068628 | 0.4384276 | up | **1.7598833** | 3.38670736 | 6.78E-05 | up | **-1.62148** | 3.07690521 | 0.00016911 | down |
| NM_001098627 | Hs.521181 | 0.18458 | 1.13648607 | 0.4597571 | up | 0.227775 | 1.17102754 | 0.3679049 | up | -0.043195 | 1.03039322 | 0.8713139 | down |
| NM_006908 | Hs.413812 | 0.33499833 | 1.26137595 | 0.7407821 | up | **2.4738283** | 5.55515949 | 0.04549342 | up | -2.13883 | 4.40404741 | 0.09281958 | down |
| NM_001197122 | Hs.75254 | 0.26039 | 1.19780246 | 0.2910272 | up | 0 | 1 | 1 | down | 0.26039 | 1.19780246 | 0.3310065 | up |
| NM_001197122 | Hs.75254 | 0.12087333 | 1.08739292 | 0.5853786 | up | -0.28454667 | 1.21802747 | 0.2256499 | down | 0.40542 | 1.32447444 | 0.1306746 | up |
| NM_015093 | Hs.269775 | 0.31235833 | 1.24173587 | 0.1570262 | up | 0.51439333 | 1.42839336 | 0.03876474 | up | -0.202035 | 1.1503198 | 0.3743523 | down |
| NM_001080124 | Hs.599762 | 0.44486333 | 1.36118517 | 0.2829594 | up | **1.3682683** | 2.5816051 | 0.01218896 | up | -0.923405 | 1.89658627 | 0.06879702 | down |
| NM_001080124 | Hs.599762 | 0.11091333 | 1.07991168 | 0.5549213 | up | 0.55537333 | 1.46954887 | 0.02213918 | up | -0.44446 | 1.36080467 | 0.06480148 | down |
| NM_001197122 | Hs.75254 | 0.68377167 | 1.60633374 | 0.319117 | up | 1.05524667 | 2.07807348 | 0.1462814 | up | -0.371475 | 1.2936748 | 0.6084944 | down |
| NM_001626 | Hs.631535 | 0.194825 | 1.14458531 | 0.1487763 | up | 0.02195 | 1.01533091 | 0.8569586 | up | 0.172875 | 1.12730273 | 0.2272186 | up |
| NM_001626 | Hs.631535 | 0.585585 | 1.50064737 | 0.03028228 | up | -0.108925 | 1.07842437 | 0.6117903 | down | 0.69451 | 1.61833469 | 0.02258195 | up |
| NM_015093 | Hs.712993 | -0.06053833 | 1.04285482 | 0.8452468 | down | -0.69341833 | 1.61711058 | 0.06106687 | down | 0.63288 | 1.55065743 | 0.1027053 | up |
| NM_001626 | Hs.631535 | -0.11045333 | 1.07956741 | 0.3383741 | down | -0.13317333 | 1.09670334 | 0.2571028 | down | 0.02272 | 1.01587296 | 0.8510885 | up |
| NM_014294 | Hs.699905 | -1.09564 | 2.13707864 | 0.06171658 | down | **-1.74677** | 3.35606347 | 0.01131618 | down | 0.65113 | 1.57039774 | 0.2558847 | up |
| NM_005465 | Hs.709089 | -0.10454333 | 1.07515401 | 0.58046 | down | -0.30134333 | 1.2322913 | 0.1457439 | down | 0.1968 | 1.14615328 | 0.3552527 | up |
| NM_005465 | Hs.498292 | -0.04224333 | 1.02971375 | 0.8314635 | down | -0.26096833 | 1.19828272 | 0.221143 | down | 0.218725 | 1.16370469 | 0.3354295 | up |
| NM_005465 | Hs.498292 | -0.448265 | 1.36439843 | 0.1848924 | down | -0.700965 | 1.62559177 | 0.05918406 | down | 0.2527 | 1.1914348 | 0.4680155 | up |
| NM_001039661 | Hs.537126 | -0.35798 | 1.28163016 | 0.04469341 | down | -0.35798 | 1.28163016 | 0.04469341 | down | 0 | 1 | 1 | down |
| NM_001039661 | Hs.537126 | -0.33723333 | 1.26333157 | 0.1045853 | down | -0.19082333 | 1.14141493 | 0.3180421 | down | -0.14641 | 1.10681185 | 0.4737968 | down |
| NM_001626 | Hs.631535 | -0.18546 | 1.1371795 | 0.3714092 | down | -0.330995 | 1.25788061 | 0.1374095 | down | 0.145535 | 1.10614077 | 0.5142639 | up |
| NM_003824 | Hs.86131 | -0.03306 | 1.02318002 | 0.7034058 | down | 0.029835 | 1.02089536 | 0.7308224 | up | -0.062895 | 1.04455974 | 0.5142673 | down |
| NM_001172566 | Hs.82116 | 0.44491333 | 1.36123234 | 0.06044027 | up | 0.21574333 | 1.16130211 | 0.3011827 | up | 0.22917 | 1.1721604 | 0.3146732 | up |
| NM_001626 | Hs.631535 | 0.01109333 | 1.00771895 | 0.9277288 | up | 0.04557833 | 1.03209683 | 0.7111307 | up | -0.034485 | 1.02419115 | 0.7974297 | down |
| NM_001098627 | Hs.521181 | 0.01102 | 1.00766773 | 0.9387366 | up | -0.068945 | 1.04894934 | 0.6342698 | down | 0.079965 | 1.0569924 | 0.6149409 | up |
| NM_001080124 | Hs.599762 | -1.46855333 | 2.76744248 | 0.1262718 | down | -1.49686333 | 2.82228432 | 0.1205247 | down | 0.02831 | 1.01981679 | 0.9758664 | up |
| NM_001080124 | Hs.599762 | -1.46891833 | 2.76814273 | 0.1032475 | down | -1.33503333 | 2.52281311 | 0.1310564 | down | -0.133885 | 1.09724447 | 0.8769941 | down |
| NM_017442 | Hs.436439 | 0.0054 | 1.00375001 | 0.9781116 | up | 0.03213 | 1.02252066 | 0.870485 | up | -0.02673 | 1.01870053 | 0.9014027 | down |
| NM_016562 | Hs.659215 | -0.29333 | 1.22546561 | 0.1971875 | down | -0.11795 | 1.08519176 | 0.5790133 | down | -0.17538 | 1.12926181 | 0.4568379 | down |
| NM_016562 | Hs.659215 | -0.254285 | 1.19274447 | 0.5950585 | down | -0.6143 | 1.53081506 | 0.2260173 | down | 0.360015 | 1.28343924 | 0.4959421 | up |
| NM_003264 | Hs.519033 | 0.80068333 | 1.741926 | 0.3178513 | up | -0.04640167 | 1.03268601 | 0.9515283 | down | 0.847085 | 1.79886261 | 0.3332174 | up |
| NM_003264 | Hs.519033 | 0.70150667 | 1.62620222 | 0.4143514 | up | -0.10921833 | 1.07864366 | 0.8956279 | down | 0.810725 | 1.75409271 | 0.3907999 | up |
| NM_138554 | Hs.174312 | 0.33798333 | 1.2639885 | 0.5933509 | up | 0.45273333 | 1.36863081 | 0.4790964 | up | -0.11475 | 1.0827874 | 0.866911 | down |
| NM_138554 | Hs.174312 | 0.83307 | 1.78147224 | 0.3118819 | up | -0.29761 | 1.22910656 | 0.7059523 | down | 1.13068 | 2.18961921 | 0.2211599 | up |
| NM_138554 | Hs.174312 | 1.048225 | 2.06798397 | 0.1837026 | up | 1.555875 | 2.94011992 | 0.06868816 | up | -0.50765 | 1.42173245 | 0.5290413 | down |
| NM_003264 | Hs.519033 | 0.69204167 | 1.61556821 | 0.3210821 | up | 0.45622667 | 1.37194882 | 0.5018196 | up | 0.235815 | 1.17757178 | 0.7472055 | up |
| NM_003263 | Hs.621817 | **1.4214783** | 2.67859847 | 0.03300775 | up | 0.55886833 | 1.47311324 | 0.3131724 | up | 0.86261 | 1.81832489 | 0.1731446 | up |

Supplementary Table S2. Correlations between the percentages of inflammatory cells staining for the toll-like receptors (TLRs) and inflammatory cell grades or percentages, such as CD4, CD8, CD68, CXCL9, CXCL10, CXCL11, and CXCR3 in skin inflammation of adult-onset Still’s disease.

| Immunohistochemical stain | Correlation coefficient, r (p-value) | | | | |
| --- | --- | --- | --- | --- | --- |
|  | TLR1 | TLR2 | TLR4 | TLR7 | TLR9 |
| CD4 | 0.126 (0.558) | 0.111 (0.606) | 0.553 (0.005) | -0.210 (0.325) | 0.147 (0.493) |
| CD8 | 0.123 (0.565) | 0.095 (0.660) | 0.114 (0.595) | -0.428 (0.037) | -0.074 (0.730) |
| CD68 | -0.202 (0.343) | 0.190 (0.373) | 0.105 (0.624) | -0.160 (0.457) | -0.153 (0.475) |
| CXCL9 | 0.067 (0.755) | 0.209 (0.327) | 0.243 (0.252) | -0.238 (0.262) | -0.135 (0.528) |
| CXCL10 | 0.014 (0.947) | 0.467 (0.021) | 0.047 (0.926) | -0.055 (0.800) | 0.285 (0.176) |
| CXCL11 | 0.444 (0.030) | 0.373 (0.072) | 0.619 (0.001) | -0.026 (0.905) | 0.456 (0.025) |
| CXCR3 | 0.346 (0.097) | 0.125 (0.562) | 0.602 (0.002) | -0.303 (0.151) | 0.318 (0.130) |
| CXCL12 | 0.204 (0.279) | 0.352 (0.056) | 0.243 (0.196) | 0.019 (0.919) | 0.540 (0.002) |
| CXCR4 | 0.052 (0.786) | -0.017 (0.928) | 0.171 (0.366) | 0.035 (0.854) | 0.334 (0.072) |

C-X-C motif chemokine 9, (CXCL9); C-X-C chemokine receptor type 3, CXCR3; toll-like receptor, TLR.
